# A comparison of student evaluations of teaching and learning in the inverted classroom model versus traditional lectures in dental education

**DOI:** 10.1186/s12909-026-09733-3

**Published:** 2026-06-20

**Authors:** Ann Katrin Reuter, James Deschner, Laura Schütz, Lisa Morlock

**Affiliations:** 1 Department of Periodontology and Operative Dentistry, University Medical Centre, Johannes Gutenberg University, Mainz, 55131 Germany; 2 Research Center for Immunotherapy (FZI), Johannes Gutenberg University, Mainz, 55131 Germany

**Keywords:** Inverted classroom model, Frontal teaching, Dental education, Learning strategies

## Abstract

**Introduction:**

The inverted classroom model (ICM) is a learner-centred approach that aims to promote engagement, independence and deeper understanding by shifting knowledge acquisition to self-directed learning before class and using class time for interactive exercises. Despite its growing popularity, there are differing views on how dental students experience and evaluate this model compared to the traditional lecture-based teaching method (FT). This study examines students’ subjective perceptions of teaching and learning within the ICM framework and investigates how a series of lectures delivered in this format influences their learning strategies and their interest in the subject area.

**Methods:**

First-year students were recruited and divided into FT and ICM groups. From April 2023 to February 2024, dental students (*n* = 138) were included in this study. The control group received classes based on the traditional (FT) concept, while the ICM group was defined as the intervention group. Data were collected at the beginning and end of the year using standardised questionnaires to evaluate the teaching concept (FLIPPY), study interest (FSI), and learning strategy (LIST-K). Wilcoxon and Mann–Whitney U tests were employed to evaluate these data, with a significance level of *p* < 0.05.

**Results:**

ICM was associated with significantly higher student ratings across most domains of the teaching concept, particularly with regard to preparation (*p* < 0.001), flexibility (*p* = 0.014), collaborative learning (*p* = 0.002), interaction with teachers (*p* < 0.001), active participation and motivation (*p* = 0.004). No significant changes in study interest or learning strategies could be observed. However, ICM students demonstrated a greater openness to innovative and digital teaching methods.

**Conclusion:**

The ICM was associated with increased student engagement and interaction. However, it was not associated with changes in study interest, learning strategies, or short-term perceived learning outcomes. The results suggest the ICM’s complementary role in blended learning but should be interpreted as associative due to the quasi-experimental design. Limitations include subjective measures, the short duration of the study, and limited generalizability.

**Supplementary Information:**

The online version contains supplementary material available at 10.1186/s12909-026-09733-3.

## Introduction

Teaching at university level, particularly in medical disciplines, presents the challenge of conveying large volumes of complex knowledge effectively. Traditionally, classroom teaching has been the dominant approach, enabling knowledge to be transferred efficiently to large numbers of students [1, 2–6]. However, this teacher-centred approach has come under increasing criticism for encouraging student passivity and limited interaction. This can result in a lack of attention, boredom and an insufficient development of critical thinking and independent problem-solving skills [1, 2, 5–8]. In response, the concept of blended learning has emerged, combining traditional and digital teaching methods. One of the best-known examples is the Inverted Classroom Model (ICM), also known as flipped classroom [9]. In the ICM, students independently access course content before class, usually using digital materials. Classroom time is then devoted to interactive and collaborative activities that consolidate and enhance understanding [9–13]. This learner-centred approach is designed to promote autonomy, engagement, and improve the quality of in-person learning [9, 10, 12–16].

Studies from various dental disciplines and educational settings have shown that ICM can improve academic performance, student motivation and engagement in class, as well as clinical skills [17–26]. For example, recent studies report improved knowledge acquisition, higher student satisfaction and enhanced practical performance in clinical training settings in the fields of orthodontics and paediatric dentistry. Furthermore, extended or hybrid ICM formats have been associated with better integration of theoretical knowledge and clinical reasoning skills [17–27].Despite this considerable evidence, the findings are not entirely consistent. Some studies highlight challenges such as an increased workload, greater cognitive demands and the need to adapt to self-directed learning, particularly in the early stages of dental education [18, 23, 26]. Furthermore, many studies focus primarily on objective outcomes, such as examination performance, whilst fewer studies systematically and multidimensionally address students’ subjective perceptions of teaching and learning processes [17, 20, 22, 25–27].

This study is based on the theoretical concepts of Bloom’s taxonomy and the ICAP framework [28–30]. ICM’s interactive face-to-face sessions address learning objectives at levels five (evaluate) and six (create) of Bloom’s taxonomy, whereas the FT achieves lower levels [28].The ICAP framework (Interactive, Constructive, Active, Passive) posits that students engage more deeply and develop stronger learning strategies when teaching activities involve constructive or interactive forms of learning rather than passive reception [29, 30]. Based on ICAP, we hypothesized that ICM would produce higher ratings on FLIPPY items measuring active and interactive engagement (e.g., collaborative learning, active participation), while no differences were expected on passive reception items [29].

This study compares FT with the ICM in dental education. The aim is to supplement the existing findings on the comparison of ICM and FT by focusing on an objective assessment of the two teaching approaches through students’ subjective perceptions. The focus is on evaluating the teaching and learning process, as well as the direct consequences for students, such as the impact on their interest in the subject and their learning strategies. The central research question is whether, from the students’ perspective, courses delivered in the ICM format are rated more highly than those delivered in the FT format, based on an evaluation questionnaire (FLIPPY). The secondary questions relate to changes in students’ academic interests and learning strategies resulting from attendance at ICM teaching sessions, as compared to FT, using the FSI and LIST questionnaires.

## Materials and methods

### Study group

The study group consisted of first-year dental students at Johannes Gutenberg University in Mainz, Germany, who attended the ‘Dental Propaedeutic with a Focus on Preventive Dentistry’ lecture from April 2023 to February 2024. Students (*n* = 83) who attended lectures based on frontal teaching principles served as the control group (CG). The other group of students (*n* = 55) served as the intervention group (IG). For this group, the lectures were delivered by the same two lecturers using the ICM concept. The difference in group sizes resulted from natural cohort allocation based on existing course structures and scheduling. An a priori power analysis was conducted using G*Power (Version 3.1.9.7; 2007). Assuming a medium effect size (d = 0.50), a significance level of α = 0.05, and a desired power of 0.80, the analysis indicated a required total sample size of *N* = 54. The final sample comprised 55 participants in the intervention group and 83 in the control group, thereby exceeding this requirement. Accordingly, the study can be considered adequately powered overall. Nevertheless, non-significant findings should be interpreted with caution, particularly in light of the unequal group sizes and their potential impact on statistical sensitivity.

The observation periods for each group were twelve weeks. The study was previously submitted to the Ethics Committee of the Rhineland-Palatinate Medical Association and received a positive vote (2023–17016). All participants were fully informed and participated in the study voluntarily.

### Frontal teaching

The CG participated in traditional face-to-face teaching in the form of a weekly 45-minute lecture. Two dentists with teaching experience took turns presenting the specified topics. The content was presented digitally using Microsoft PowerPoint. Students were given the opportunity to ask questions about the content directly on site. To support review and consolidation, lecture slides were made available after each session via the university’s internal, password-protected Moodle learning platform (Moodle – Central Learning Management System, Johannes Gutenberg University Mainz, Germany). This digital provision was limited to passive access to materials and did not include interactive or preparatory online components. For this reason, the CG’s teaching approach is regarded as traditional, teacher-centred instruction and is not a hybrid or blended learning model.

### Inverted classroom model

The IG was taught on the basis of the ICM, with a focus on active and interactive learning. The weekly teaching unit was divided into two separate phases:

#### Digital asynchronous preparation (15 min)

Two dentists recorded the theoretical content on video using Open Broadcaster Software Studio (Open Broadcaster Studio Contributors, San Francisco/USA). These 15-minute digital, asynchronous lectures were made available to students via the password-protected learning Moodle platform one week before the face-to-face session.

Students’ main task in this phase was to prepare independently for the upcoming teaching unit. Participants were encouraged to note down any questions or areas of confusion that arose while watching the videos. The corresponding lecture slides were also made available.

#### Interactive face-to-face session (30 min)

Building on the digital preparation, an interactive teaching session lasting 30 min and using various interactive learning methods was held in person. The primary purpose of this session was to consolidate the prepared content, with the lecturers shifting their role to moderation, answering questions and addressing any issues students had with comprehension. The intention was to use the face-to-face time for active learning like for example mind-mapping, quizzes and scale questions.

### Questionnaires

To evaluate the two teaching concepts, the students of both groups completed standardized and validated questionnaires [31–35]. In order to record changes in study interest and the personal learning strategies, students of the CG und IG completed the corresponding FSI and LIST questionnaires at the beginning of the year (t0) and twelve weeks later (t1). To compare the evaluation of the inverted classroom concept and traditional classroom teaching, both groups completed the FLIPPY questionnaire at the end of the semester (t1), with the IG also having to answer specific questions on the inverted classroom concept. All questionnaires were created using the EvaSys (electric paper evaluation systems) survey software and implemented in coordination with the Center for Quality Assurance & Development (ZQ) at Johannes Gutenberg University Mainz. The students completed the questionnaires online using a QR code or link, with the data being collected electronically directly via the EvaSys portal. A personal code generated from the students’ and their parents’ dates of birth was used to compare the data over the longitudinal period. This enabled the responses from t0 and t1 to be matched while maintaining pseudonymity.

#### Sociodemographic parameters

The following socio-demographic parameters were collected in the questionnaires: Age, gender, native language, semester, previous education and professional activity alongside studies.

#### Measuring interest in studying and learning strategies (pre-post measurement)

Two established instruments were used in the first and second surveys to record changes in student characteristics.

##### Questionnaire on interest in studying (FSI)

This instrument uses 18 items across three components to measure increases in study interest: interest, emotional valence and value-related valence. Responses were given on a four-point Likert scale ranging from 0 (“does not apply at all”) to 3 (“applies completely”) [33].

##### Questionnaire on learning strategies in studies – short scale (LIST-K)

The Questionnaire on Learning Strategies in Studies – Short Scale (LIST-K) was also used [35]. This shortened version, comprising 39 items across 13 subscales, measures changes in learning strategies relating to cognitive and metacognitive processes, as well as strategies for managing internal and external resources. Students used a five-point Likert scale (1 = “very rarely” to 5 = “very often”) [35].

In addition, the surveys included five questions on personal attitudes towards teaching that had been validated in a pre-test.

#### Evaluation of the teaching concept (post-measurement)

##### Questionnaire for evaluating flipped classroom courses (FLIPPY)

The second survey involved a detailed evaluation of the respective teaching concept, included the standardised, adapted FLIPPY questionnaire, as well as the additional supplementary questions.

The FLIPPY questionnaire for Evaluating Flipped Classroom Courses (Version 0.2) was used [32]. This version consists of 22 items in five aspects (preparation, learning experience, flexibility, collaborative learning and teacher). It uses a five-point Likert scale ranging from 1 (“strongly disagree”) to 5 (“strongly agree”) [32]. A sixth aspect (videos) has been added to the ICM group comprising six more points for evaluating digital asynchronous lectures [32].

##### Additional questions on active participation and motivation

Seven additional questions were added to the active participation and motivation aspect to deepen the evaluation, based on the BEvaKomp [34], FLIPPY Version 0.1 [31] and Inventory for evaluating blended learning (IEBL) [36] instruments. These questions were also answered using a five-point Likert scale.

### Statistical analysis

Statistical analysis and data presentation were carried out using SPSS Statistics version 29 (IBM, Armonk, New York, USA) and Excel version 2024 (Microsoft, Redmond, Washington, USA), under the supervision of the Institute of Medical Biometry, Epidemiology and Informatics (IMBEI) at the University Medical Centre Mainz. The socio-demographic parameters of the students were evaluated descriptively using frequency analysis.

The Wilcoxon rank sum test was used to analyse intra-individual differences at the beginning of the semester (t0) and at the end of the semester (t1) in the FSI-, LIST- questionnaires and personal attitude towards study, as no normal distribution could be assumed. Both the Shapiro-Wilk and the Kolmogorov-Smirnov tests confirmed that the data were not normally distributed (*p* < 0.001). The Mann–Whitney-U test for unpaired samples was used to compare the intervention and control groups’ results. In addition to the exploratory testing, Bonferroni–Holm correction was applied to determine the adjusted *p*-values for the confirmatory analysis of the hypotheses.

The significance level was set at *p* < 0.05 for all comparisons. Rosenthal’s r was used to calculate effect sizes. According to Cohen’s conventions, values of *r* ≈ 0.1 indicate a small effect, *r* ≈ 0.3 a medium effect, and *r* ≥ 0.5 a large effect. In addition, Cronbach’s alpha was used to assess the internal consistency of the questionnaire items.

No pattern could be identified in the missing data, which is why we assume that the data is missing entirely at random. Missing data were handled using listwise deletion. Cases were excluded due to duplicate questionnaire submissions, incomplete responses, or inability to match the personal code. No systematic differences between included and excluded students were identified.

## Results

### Socio-demographic parameters

A total of 148 students took part in the study. Ten students were excluded due to missing data, 138 students were included. The IG comprised 55 students, while the CG consisted of 83 students (Table 1). A statistical comparison of the socio-demographic variables of the two groups was carried out, and no significant differences were found (supplementary file 1).

**Table 1:** Socio-demographic parameters of the students

Feature	Characteristic	Number
Age	years	21.01 (SD ± 2,452)
Gender	male	36
	female	102
	divers	0
Mother tongue	German	113
	other	25
Previous education	medical/dental/dental technician training	31
	university degree	7
	none	92
	other	8
Professional activity alongside studies	medical/dental/dental technology sector	42
	non-medical sector	28
	none	68

### Questionnaire for evaluating inverted classroom courses (FLIPPY)

Due to the difference in group sizes (IG: 55 students, CG: 83 students), a Mann-Whitney U test was carried out at time t0. The results showed that there was no significant difference between the two groups (*p* = 0.821). All *p*-values for the scores are adjusted *p*-values, adjusted using the Bonferroni-Holm method.

#### Preparation

**Fig. 1:**
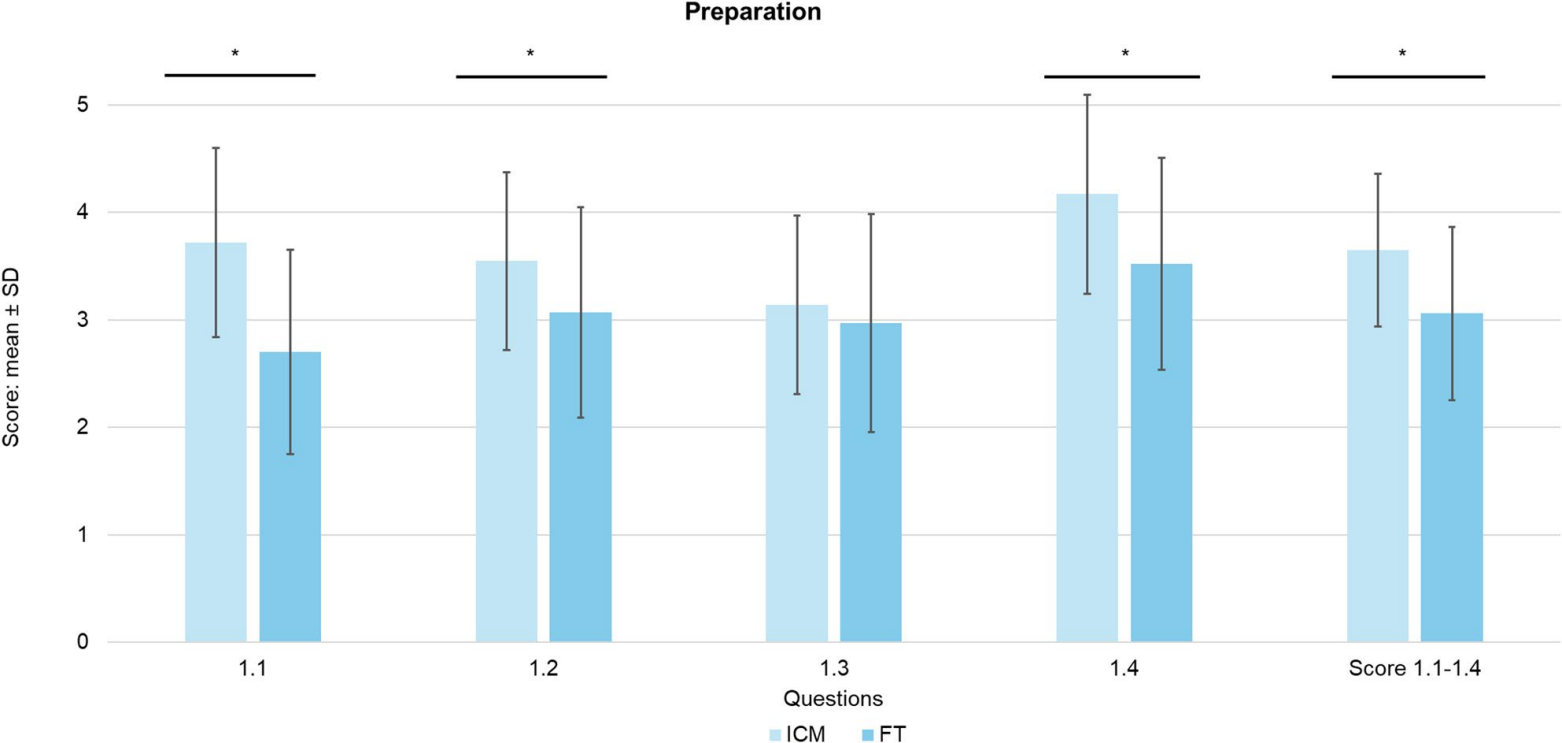
Mean value comparison of ICM vs. FT for Q 1.1–1.4, preparation (*significant *p* < 0.05); 1.1: I am regularly preparing for a course. 1.2: I am putting a lot of effort into preparing for the course. 1.3: I enjoy preparing for the course. 1.4: I found the course preparation useful

When comparing the preparation aspect IG students reported significantly higher scores than CG for Question (Q) 1.1 (*p* < 0.001; *r* = 0.56, according to Cohen’s conventions for r, values of 0.56 represent large effects) on regular preparation for the event (Fig. 1).

Similarly, Q 1.2 (*p* = 0.008; *r* = 0.49, according to Cohen’s conventions for r, values of 0.49 represent medium effects) on the effort involved in preparing for the event and Q 1.4 (*p* = 0.016, *r* = 0.46 according to Cohen’s conventions for r, values of 0.46 represent medium effects) on the usefulness of preparing for the event were rated more highly by the IG. The overall preparation score was significantly higher in IG than CG (*p* = 0.016; *r* = 0.55, according to Cohen’s conventions for r, values of 0.55 represent large effects) (Fig. 1). The Cronbach’s alpha coefficient for the preparation is 0.835.

#### Learning experience

**Fig. 2:**
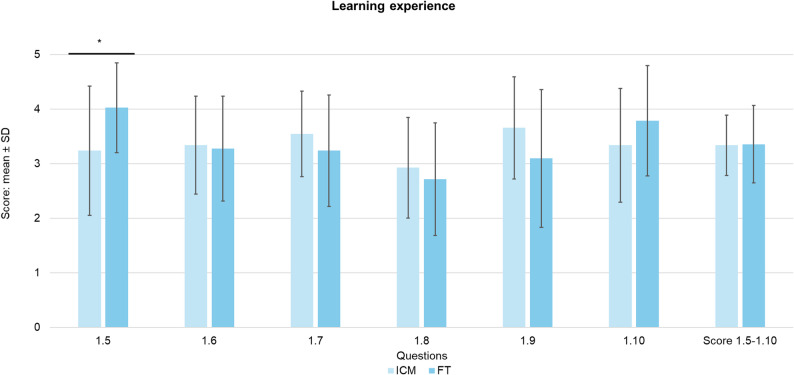
Mean value comparison of ICM vs. FT for Q 1.5–1.10, learning experience, (*significant *p* < 0.05). 1.5: I feel like I learned a lot of new things at this course. 1.6: I feel adequately prepared for the examination thanks to the course. 1.7: I gained important transfer knowledge and a deeper understanding through the course. 1.8: I learn superficially and by heart in the course. 1.9: I find it difficult to follow the course continuously. 1.10: I feel that I can effectively acquire knowledge in this course

For perceived learning experience only Q 1.5 (acquisition of new knowledge) was significantly higher in CG than IG (*p* = 0.013; *r* = 0.47, according to Cohen’s conventions for r, values of 0.47 represent medium effects). No other items differed significantly (Fig. 2). The Cronbach’s alpha coefficient for the learning experience is 0.542 (low internal consistency) (supplementary file 2).

#### Flexibility

**Fig. 3:**
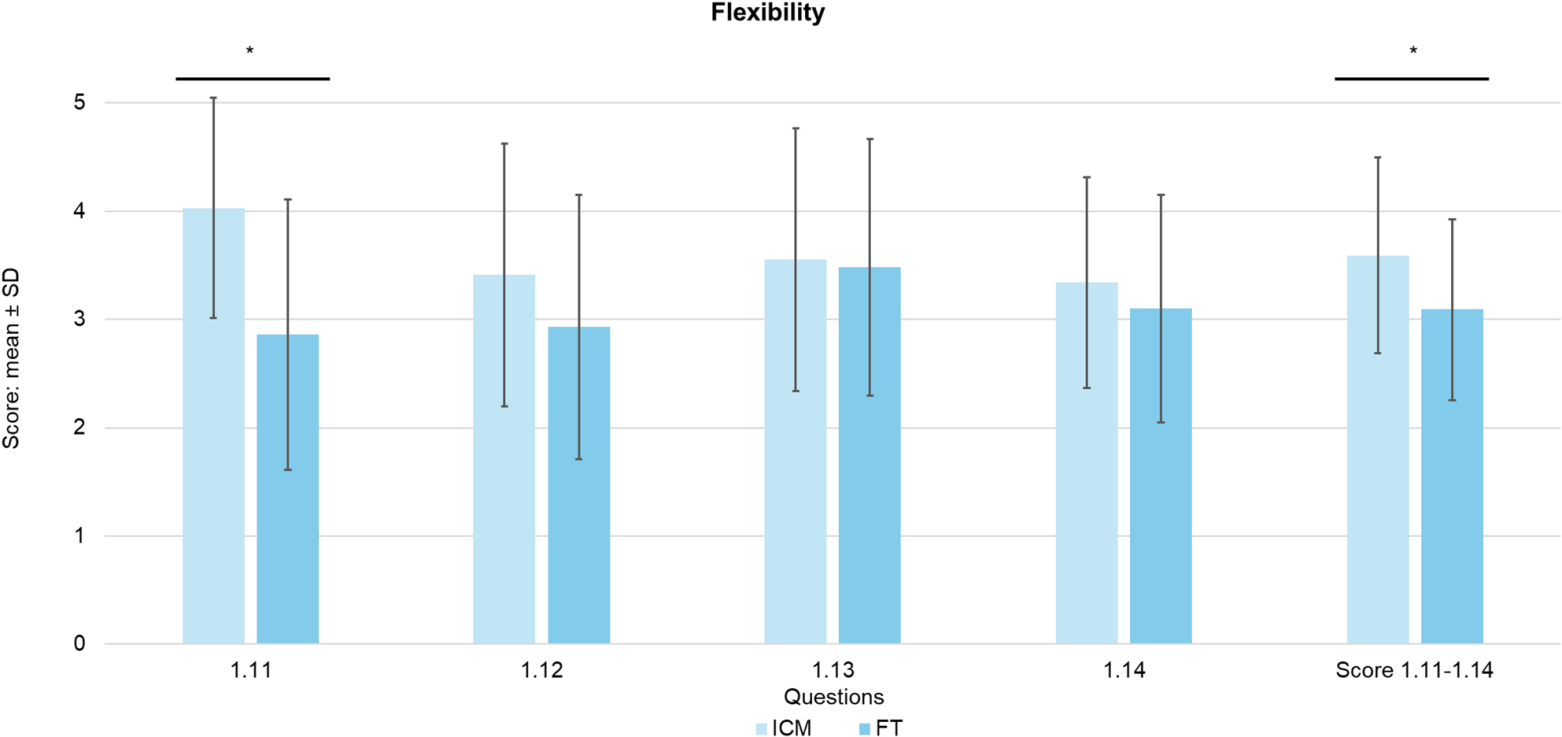
Mean value comparison of ICM vs. FT for Q 1.11–1.14, flexibility (*significant *p* < 0.05); 1.11: I can acquire learning content at a time that suits me. 1.12: I feel like I can determine my own learning pace. 1.13: My learning activities are easily compatible with my private life. 1.14: The course offers me the opportunity to apply different learning styles

IG students rated flexible learning times Q1.11 significantly higher (*p* = 0.005; *r* = 0.47, according to Cohen’s conventions for r, values of 0.47 represent medium effects) and achieved a significantly higher overall flexibility score (*p* = 0.028; *r* = 0.46, according to Cohen’s conventions for r, values of 0.46 represent medium effects). Items Q1.12–1.14 showed non-significant trends favouring IG (Fig. 3). The Cronbach’s alpha coefficient for the flexibility is 0.734.

#### Collaborative learning

**Fig. 4:**
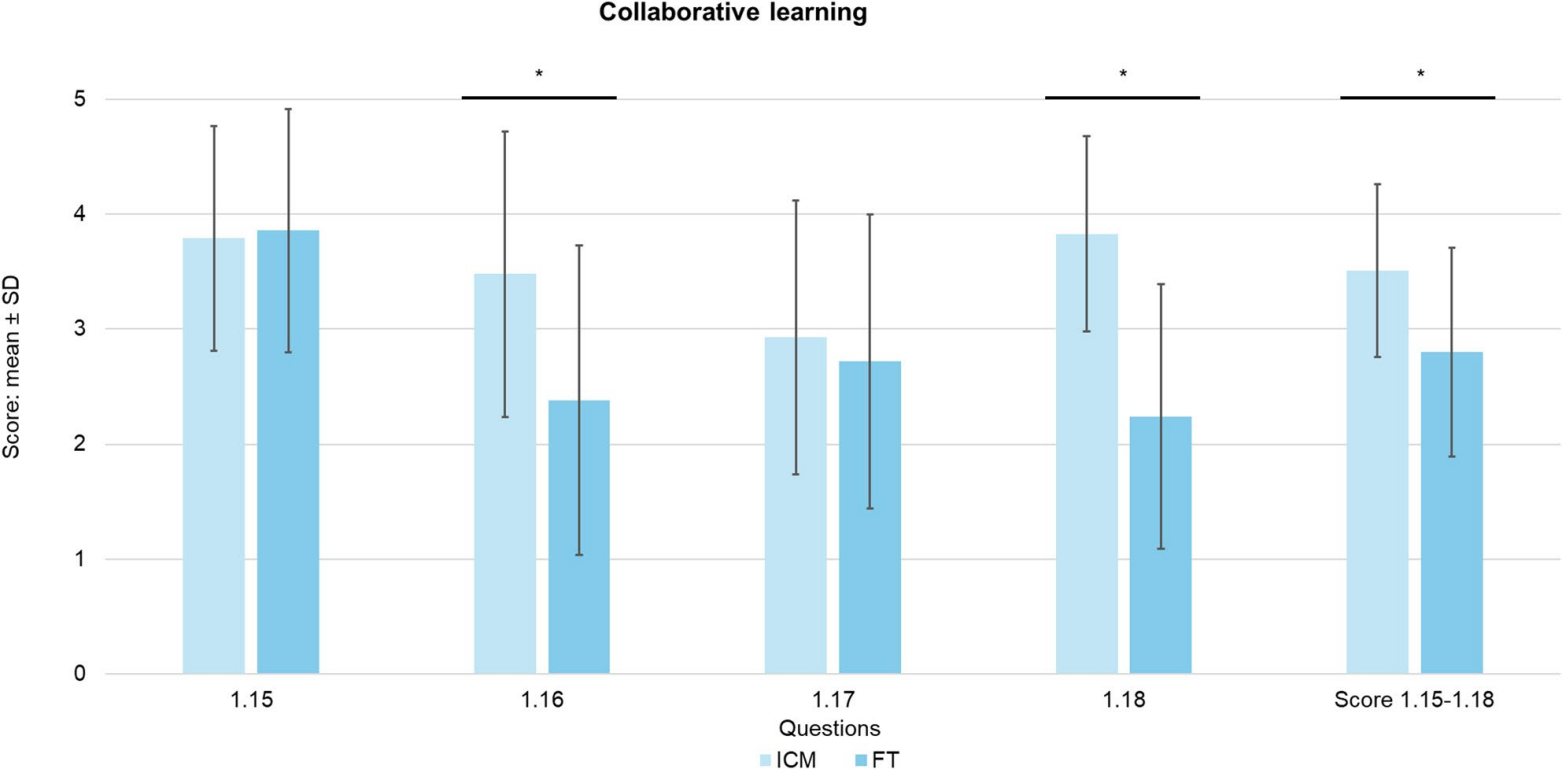
Mean value comparison of ICM vs. FT for Q 1.15–1.18, collaborative learning, (*significant *p* < 0.05); 1.15: It is quite possible to interact with other students. 1.16: The learning content is meaningfully reinforced through group/partner work. 1.17: Group work and partner work help me to understand certain topics better. 1.18: The event provides many opportunities for interaction with other students

IG students rated collaborative learning significantly higher than CG for Q 1.16 (*p* = 0.007; *r* = 0.51, according to Cohen’s conventions for r, values of 0.51 represent large effects) on the usefulness of group work and Q 1.18 (*p* < 0.001; *r* = 0.64, according to Cohen’s conventions for r, values of 0.64 represent large effects) on the opportunity for exchange with others. Overall collaborative learning scores were significantly higher in IG (*p* = 0.01; *r* = 0.58, according to Cohen’s conventions for r, values of 0.58 represent large effects) (Fig. 4). The Cronbach’s alpha coefficient for the collaborative learning is 0.686.

#### Teacher

**Fig. 5:**
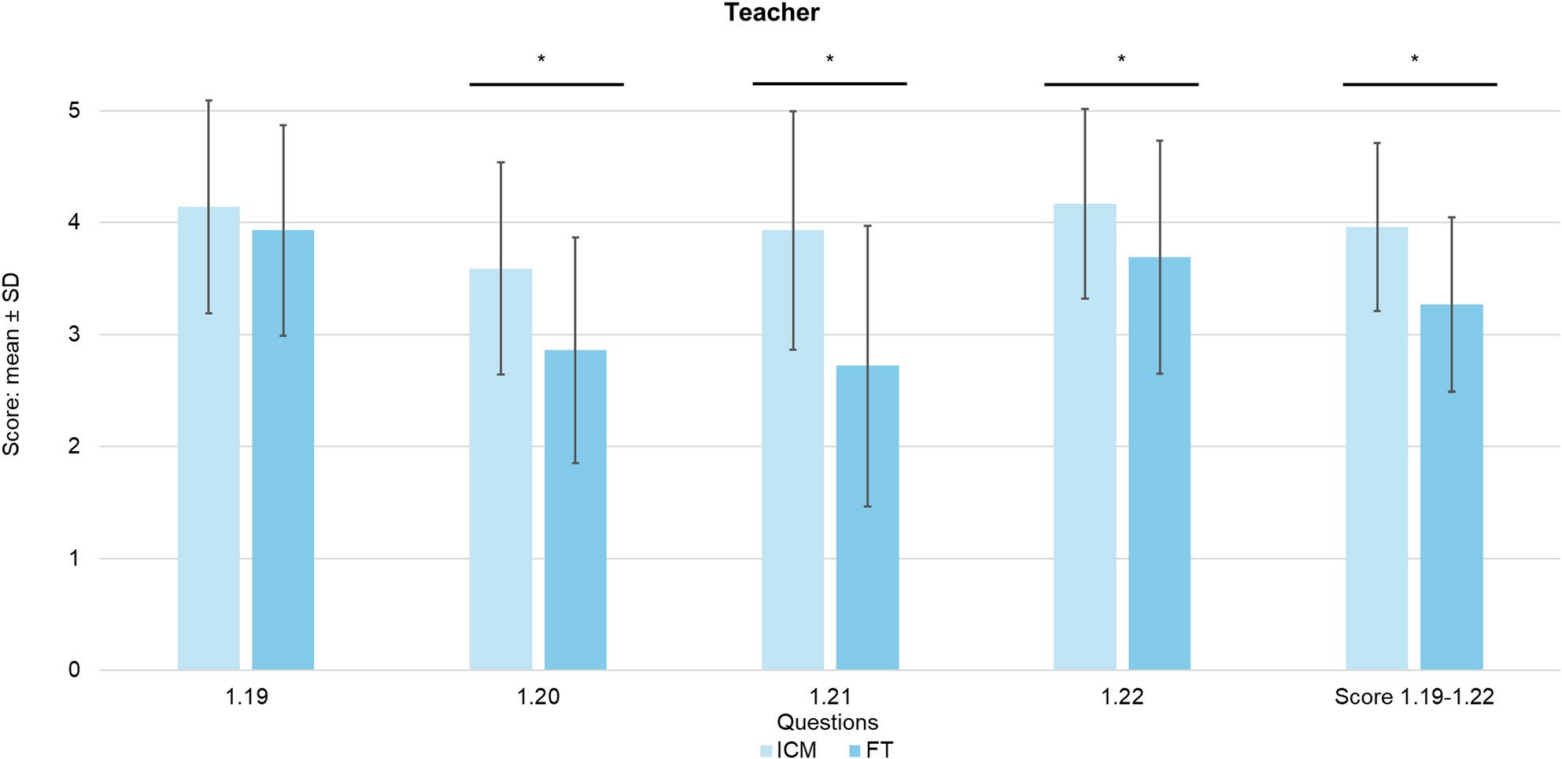
Mean value comparison of ICM vs. FT for Q 1.19–1.22, teacher, (*significant *p* < 0.05); 1.19: The teacher is cooperative and open-minded., 1.20: The teacher encourages students to critically examine the content of the course. 1.21: The teacher encourages questions and active participation. 1.22: It is important to the teacher that the students learn something

IG students rated the teacher significantly higher for Q 1.20 on encouraging critical engagement with the course content (*p* < 0.001; *r* = 0.63, according to Cohen’s conventions for r, values of 0.63 represent large effects), Q 1.21 on promoting questions and active participation (*p* = 0.010; *r* = 0.61, according to Cohen’s conventions for r, values of 0.61 represent large effects) and Q 1.22 on the importance of student-centred learning (*p* = 0.010; *r* = 0.48, according to Cohen’s conventions for r, values of 0.48 represent medium effects). In addition, the resulting score was significantly higher in the IG (*p* = 0.006; *r* = 0.66, according to Cohen’s conventions for r, values of 0.66 represent large effects) (Fig. 5). The Cronbach’s alpha coefficient for the teacher is 0.821.

#### Active participation/motivation

IG students reported higher engagement in Q 1.23 on participation with verbal contributions (*p* < 0.001; *r* = 0.65, according to Cohen’s conventions for r, values of 0.65 represent large effects), Q 1.24 on expressing one’s own opinions (*p* = 0.004; *r* = 0.54, according to Cohen’s conventions for r, values of 0.54 represent large effects), Q 1.25 on asking questions (*p* = 0.002; *r* = 0.58, according to Cohen’s conventions for r, values of 0.58 represent large effects), Q 1.26 on the comprehensible formulation of verbal contributions (*p* = 0.003; *r* = 0.57, according to Cohen’s conventions for r, values of 0.57 represent large effects).

**Fig. 6:**
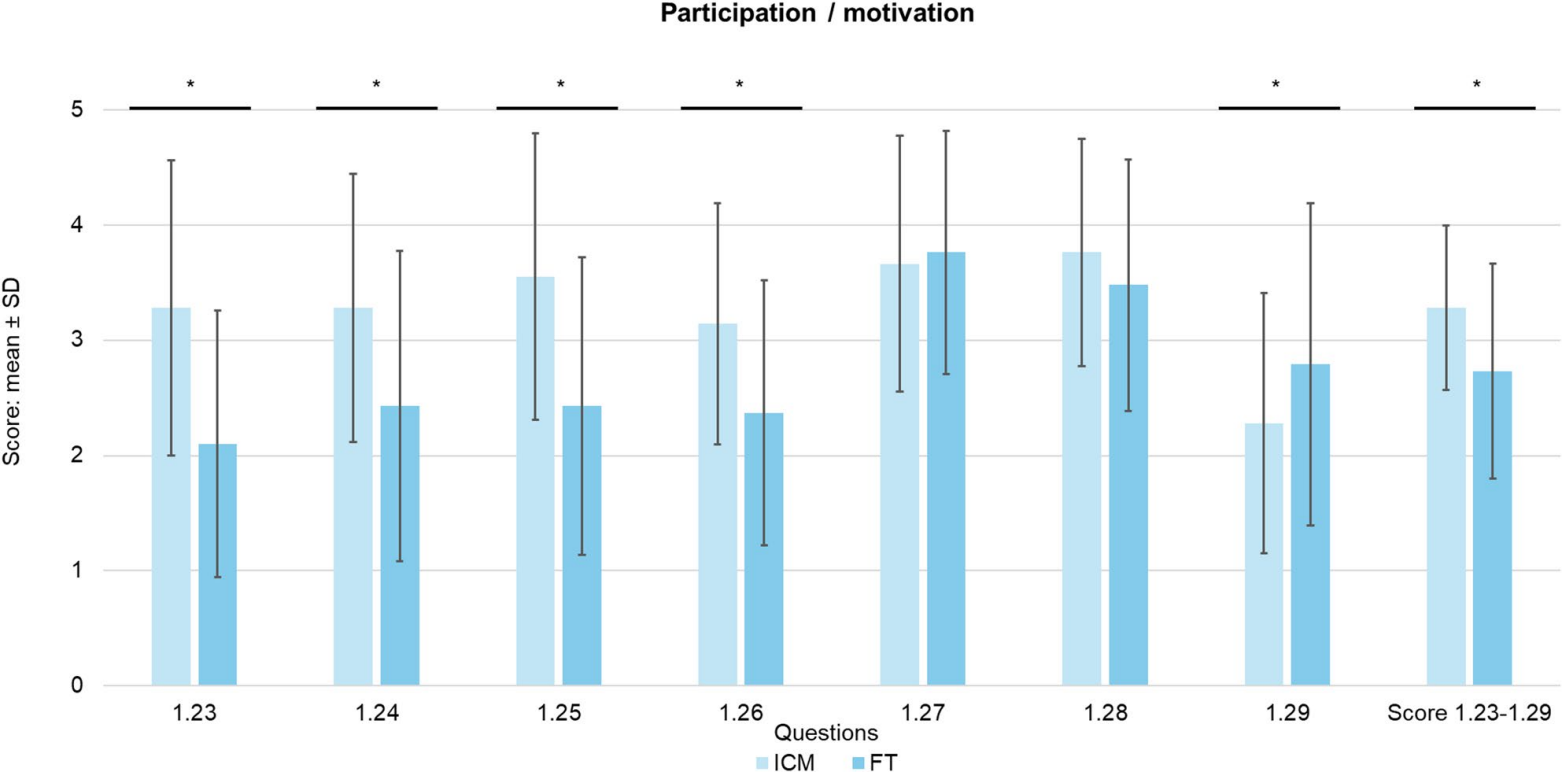
Mean value comparison of ICM vs. FT for Q 1.23–1.29, Active participation/motivation, (*significant *p* < 0.05); 1.23: I participated in this course by contributing to discussions. 1.24: This course has made it easier for me to express my own impressions/opinions. 1.25: Thanks to this course, I find it easier to ask questions when I do not understand something. 1.26: The event provides many opportunities for interaction with other students. 1.27: I prepare the content of a course well. 1.28: The course motivates participants to engage with the content themselves. 1.29: I met up with other students outside of the course to discuss the content in more depth

However, CG reported higher scores for Q 1.29 (deepening content outside class, *p* = 0.026; *r* = 0.43, according to Cohen’s conventions for r, values of 0.43 represent medium effects). Overall, the active participation/motivation score was significantly higher in IG (*p* = 0.012; *r* = 0.55, according to Cohen’s conventions for r, values of 0.55 represent large effects) (Fig. 6). The Cronbach’s alpha coefficient for the active participation/motivation is 0.786.

#### Video materials

IG rated all video-related items positively. Cronbach’s alpha is 0.746 (supplementary file 3).

### Results of the questionnaire on study interest (FSI)

Due to the difference in group sizes (IG: 55 students, CG: 83 students), a Mann-Whitney-U test was carried out at time t0 (*p* = 0.782). The results showed that there was no significant difference between the two groups.

Overall, there were no significant changes to most items. However, three items differed significantly between measurement points. Q 2.11 asked about the importance of studies compared to other valued aspects of life. IG students showed a reduced tendency to prioritize hobbies and social life over their studies (*p* = 0.046; *r* = 0.53, according to Cohen’s conventions for r, values of 0.53 represent large effects). Q 2.16 concerned enjoyment of academic content. IG significantly disagreed more with not enjoying the subject content (*p* = 0.014; *r* = 0.65, according to Cohen’s conventions for r, values of 0.65 represent large effects): Q 2.17 asks about voluntary engagement with the subject matter prior to studying. The CG showed a reduction in engagement with study content (*p* = 0.016; *r* = 0.38, according to Cohen’s conventions for r, values of 0.38 represent medium effects) (supplementary file 4). The Cronbach’s alpha for the items in the FSI questionnaire is 0.807.

### Results of the questionnaire on learning strategies during studies (LIST)

Due to the difference in group sizes (IG: 55 students, CG: 83 students), a Mann-Whitney-U test was carried out at time t0 (*p* = 0.386). The results showed that there was no significant difference between the two groups.

Most items in this section showed no statistically significant changes in either group.

However, the IG reported a reduction in memorising strategies in Q 3.12 (*p* = 0.031; *r* = 0.58, according to Cohen’s conventions for r, values of 0.58 represent large effects).The CG showed regressive changes in terms of a lack of planning in their approach to studying Q 3.15, (*p* < 0.001; *r* = 0.55, according to Cohen’s conventions for r, values of 0.55 represent large effects), difficulty concentrating and staying focused Q 3.22 (*p* = 0.026; *r* = 0.35, according to Cohen’s conventions for r, values of 0.35 represent medium effects), being unable to concentrate while studying Q 3.23 (*p* = 0.047; *r* = 0.31, according to Cohen’s conventions for r, values of 0.31 represent medium effects) and seeking additional literature. Additionally, items related to seeking supplementary information revealed significant regressive results: Q 3.34 (searching for additional literature when the content is unclear; *p* = 0.044, *r* = 0.32, according to Cohen’s conventions for r, values of 0.32 represent medium effects); Q 3.35 (gathering missing information from multiple sources; *p* = 0.029, *r* = 0.35, according to Cohen’s conventions for r, values of 0.35 represent medium effects); and Q 3.36 (using additional literature when the notes are incomplete; *p* = 0.008, *r* = 0.42, according to Cohen’s conventions for r, values of 0.42 represent medium effects). The Cronbach’s alpha for the items in the LIST questionnaire is 0.685 (supplementary file 5).

### Results of the questionnaire on personal attitude towards teaching

Most items showed no significant changes. One exception was Q 4.3, which relates to the perceived importance of and preference for in-person teaching within the study programme. This item increased in IG over time (*p* = 0.020; *r* = 0.62, according to Cohen’s conventions for r, values of 0.62 represent large effects), and the group comparison was also significant (*p* = 0.005; *r* = 0.38, according to Cohen’s conventions for r, values of 0.38 represent medium effects). Cronbach’s alpha for the items in the questionnaire on personal attitudes towards teaching is 0.174 (low internal consistency) (supplementary file 6, 7).

## Discussion

This study examined students’ perspectives on the use of ICM compared to FT. The evaluation focused on the assessment of the teaching concept and its influence on study interest, learning strategies and personal attitudes towards teaching in the preclinical phase of dental degree programmes. The central research question regarding the evaluation of the ICM teaching concept in comparison with FT is addressed through an analysis of the FLIPPY results. The sub-questions regarding the influence on academic interest and learning strategies are analysed using the results of the FSI and LIST questionnaires.

### FLIPPY questionnaire

The aspect of preparation showed significant improvements with moderate to strong effect sizes in the ICM group, both in the overall score and in scores 1.1 (regular preparation), 1.2 (effort required for preparation) and 1.4 (course preparation was useful). Students in the ICM group reported that they were preparing for lessons more regularly, enthusiastically and intensively, which can be attributed to the model’s promotion of self-discipline, personal responsibility and self-organisation [37–39]. These results are consistent with those of Gebhardt and Weber, who also demonstrated a higher quality of preparation in the ICM [40]. Although perceived enjoyment of preparation (Q 1.3) did not increase significantly, the ICM is often described in the literature as being more motivating and entertaining [41–44].

In terms of learning experience, there were no changes in favour of the ICM. Only Q 1.5 (learning a lot of new things) showed an improvement with the FT. As the internal consistency of this section of the questionnaire is very low, this effect should be interpreted with caution (supplementary file 2). In the traditional format, more material is covered per unit of time. By contrast, the ICM lays the foundation for knowledge acquisition during the self-study phase, with the classroom phase serving to reinforce this knowledge [1, 6, 9, 14, 39]. Students perceived the ICM as promoting deeper engagement and long-term knowledge retention, as emphasised by the revised Bloom’s Taxonomy of Learning Objectives [9, 37, 41, 45]. Studies show that the ICM strengthens the use of deep learning strategies and reduces superficial learning [40, 41]. Overall, despite the lack of statistical significance, students found the ICM to be more conducive to learning and better suited to exam preparation.

Students in the ICM group reported significant improvements in terms of time flexibility (Q 1.11) and the ability to adapt to different learning styles (Q 1.14).Digital learning content, such as instructional videos, enables students to learn independently of time and place, allowing them to organise their learning in a self-determined manner [10, 12, 14, 46–48]. These results are consistent with the findings of Gebhardt and Weber, and the reviews by Gianoni-Capenakas et al. and Bizhang et al., who also reported an increased perception of flexibility in the ICM [40, 43, 44].

Clear advantages for the ICM were evident in the aspect of collaborative learning. In particular, Q 1.16 (reinforcement of the learning content through group / partner work) and 1.18 (many interactions with other students) showed an improvement. Students on the ICM reported that the course encourages group work and discussion through its interactive teaching methods. This promotes higher cognitive learning levels, in accordance with the ICAP model. In line with the ICAP model, learning becomes progressively deeper as students shift from Passive to Active, Constructive, and ultimately interactive engagement [29, 30, 49, 50]. Studies by Vanka et al., Ramnanan et al., and Park et al. demonstrate that students rate the quality and scope of exchange in the ICM particularly highly [3, 41, 42]. The results confirm that the ICM improves knowledge transfer and understanding through collaborative learning processes.

Although both teachers taught both FT and ICM, consistent improvements in the teacher aspect were detected in favour of ICM in Q 1.20–1.22 (the teacher values active thinking, questioning and genuine learning). The ICM was associated with a higher quality and more intensive level of interaction between students and teachers [14, 41, 42, 51]. Within student-centred teaching concepts, such as the ICM, teachers actively promote learning processes, encourage critical engagement, and prioritise individual learning success [4, 52–54]. In the present study, this stronger focus on support resulted in teachers being perceived much more positively.

Significant improvements were also observed in terms of active participation and motivation among the ICM group. Questions 1.23–1.26 (the course has helped me participate more actively, express my opinions more clearly, ask questions, and interact with other students) showed that students in the ICM group took a more active role in discussions, asking questions more frequently and demonstrating greater motivation to explore content independently. However, Q 1.27 (follow-up on content) and 1.29 (meet other students to revise the course content) only showed advantages for the FT. These results confirm earlier studies’ findings that the ICM was associated with greater intrinsic motivation and participation in learning [9, 14, 41, 42].

The ICM-specific aspect videos were rated very positively by the students overall. They particularly appreciated the quality, comprehensibility and effectiveness of the instructional videos, as well as having the opportunity to review learning content repeatedly and at their own pace [3, 42, 46–48, 55]. These findings are consistent with those of Ramnanan et al. and Bohaty et al., who emphasise self-determined access to instructional videos as a key advantage of ICM [3, 55]. However, Park et al. identified technical requirements and potential access problems as possible challenges [41, 48].

### Study interest

Assessing the influence of the two teaching concepts on study interest across the semester indicated that only few significant changes occurred. Within the IG, differences only appeared in items 2.11 (studies being of low importance compared to private life) and 2.16 (dealing with the content of my studies being one of my least favourite activities). In the CG, however, item 2.17 (being engaged with the content of my field of study before beginning my studies) was the only one to show a significant change. No significant differences were found between the two groups. From a theoretical perspective, this absence of clear group differences in study interest, as outlined by Bloom’s Taxonomy and the ICAP framework [28, 29], may suggest that the ICM primarily operates at higher levels of cognitive engagement (e.g. application and active participation), without necessarily influencing affective-motivational constructs, such as study interest. In ICAP terms, the intervention may have shifted behavioural engagement towards “active” or “constructive” modes during learning activities, while leaving the underlying “interactive” or affective domain (as operationalised by the FSI) largely unchanged. These results can be explained by the limited application of the teaching concepts over time. Importantly, these largely non-significant findings should not simply be dismissed as inconclusive. Rather, they may suggest that implementing the ICM does not significantly impact students’ interest in the subject within the observed timeframe, or that the two teaching concepts have comparable effects on this construct. One possible explanation is ceiling effects: both groups exhibited high mean scores on many items at the beginning of the year (t0), suggesting strong baseline interest among dentistry students that left little room for improvement. It is also conceivable that the measurement instrument lacked sufficient sensitivity to detect subtle changes in study interest in this population. Alternatively, study interest may be a relatively stable trait that is less susceptible to short-term pedagogical interventions. As the ICM was employed for the first time, its impact on study interest could only become apparent following a period of adaptation to the new teaching approach [3, 43, 56]. At the beginning of the year (t0), both groups exhibited high mean scores on many items, indicating a strong fundamental interest in studying among dentistry students.

A significant change in item 2.11 (low importance of studies compared to private life) suggests that IG students placed a relatively greater emphasis on leisure activities and social relationships during the lecture period. However, this finding should be interpreted with caution, as a single item may reflect various underlying factors, such as differences in time management, reduced feelings of guilt associated with leisure time or variations in response behaviour. Similarly, item 2.16 (Dealing with the content of my studies is not one of my favourite activities) indicated that ICM students were less engaged with study-related activities during the lecture period. These developments could be related to the increased personal responsibility and higher workload in the ICM, which makes balancing studies and leisure time difficult [43, 56]. This should be viewed as a significant limitation of the ICM. When considered in relation to Cognitive Load Theory, this suggests that first-year students require support to effectively structure the learning content during the self-study phase, thereby reducing the learning-related load [57]. The engagement with the subject matter (point 2.17) decreased in the CG, which underscores the passivity of the FT.

### Learning strategies

The influence of the two teaching concepts on learning strategies resulted in only a few significant changes. While this may be partly due to the short duration of the intervention, this explanation alone is insufficient, given that significant effects were observed for FLIPPY outcomes.

From a theoretical perspective, the largely non-significant findings of the LIST-K, as informed by the ICAP framework and Bloom’s Taxonomy [28, 29], may suggest that the ICM primarily influences observable cognitive engagement processes (e.g. active participation, application, and comprehension) rather than deeper, more stable self-regulatory learning strategies. According to the ICAP framework, the intervention may therefore primarily influence “active” learning behaviours during instruction without necessarily resulting in measurable changes in internalised metacognitive or resource management strategies, as assessed by the LIST-K.

As with the findings on study interest, the largely non-significant results of the LIST-K should be considered meaningful rather than incidental. They may suggest that the ICM does not substantially alter learning strategies within the timeframe of this study or that instructional approaches yield comparable outcomes in this regard. Several explanations are plausible: the learning strategies measured by the LIST-K may be relatively stable constructs requiring longer or more intensive interventions to change; ceiling effects may have limited the observable variance; or the instrument itself may not have been sensitive enough to capture subtle, context-specific adaptations in students’ learning behaviour. Alternatively, the absence of significant differences may reflect an actual equivalence between the teaching methods with respect to learning strategies [58].

The only significant change in the IG concerned item 3.12, which is associated with the cognitive strategy of repetition. At the beginning of the year (t0), students relied more on memorising scripts and notes than they did at the end of the observation period (t1). This could suggest that, in the long term, the ICM encourages more understanding-oriented learning behaviour, with mechanical repetition decreasing in favour of more in-depth processing [3, 43, 56, 59, 60]. However, this interpretation should be treated with caution, as it is based on a single item and therefore does not allow for robust conclusions about broader changes in learning strategies.

In contrast, the CG showed several notable changes. Item 3.14 (“Goals and Planning”) suggests that students planned their learning more systematically at the start of the year. Items 3.22 and 3.23 (internal resource management) suggest a greater level of focus and attentiveness at the start of the year. Items 3.34–3.36 (external resource management) demonstrate that students used additional literature and sources more frequently in the early stages of the year.

These results suggest that there may be a decline in strategic learning activity over the course of the year regardless of the teaching concept, possibly due to increasing exam stress or the effects of fatigue [41–43].

Overall, it can be concluded that a longer longitudinal study would be necessary to identify significant differences in the development and application of learning strategies, and to determine the lasting effects of teaching concepts, especially ICM.

### Personal attitude towards teaching

The results regarding the students’ personal attitudes towards teaching showed very similar trends between the IG and CG. Over the course of the 12 weeks, a significant change was observed only in the IG for question 4.3. This significance was also confirmed in the group comparison between the IG and CG. Results related to personal attitudes towards teaching should be interpreted with caution due to very low internal consistency (Cronbach’s α = 0.174) and weak inter-item correlations. Accordingly, these items were retained for exploratory purposes only and were not considered to constitute a reliable scale (supplementary file 7).

Item 4.3 referred to the importance of, and preference for, face-to-face teaching in the dentistry program. At the start of the year (t0), students in the IG rated face-to-face teaching as important and preferred it. However, towards the end of the 12 weeks (t1), there was increasing acceptance and appreciation of digital teaching formats. Through their experiences with the ICM, which combines elements of blended learning like asynchronous digital preparation phases with interactive face-to-face sessions, the students realised that digital teaching could be successfully integrated into dental studies and be perceived as equally relevant [4, 9, 14, 40–43, 52, 56, 59, 60].

This development was supported by the mean values for question 4.5, which showed an increase in approval for the combination of digital and face-to-face teaching methods. Question 4.2 (be open to new teaching methods) also revealed that IG students had become more open to new teaching methods and innovative approaches to teaching materials by the end of the year. CG students also demonstrated a fundamentally positive attitude towards methodological innovations.

Furthermore, both groups agreed with the statement in question 4.1 that teaching should adapt to contemporary developments and advances. However, both groups rejected exclusively digital teaching (question 4.4), highlighting the ongoing importance of personal interaction and practical face-to-face components in their studies [14, 40–42].

### Strengths, limitations and generalisability

#### Strengths

A major strength of this study is the direct comparison of two teaching concepts within the same curricular context, with identical lecturers and content. Additionally, the use of validated instruments enabled a multidimensional assessment of student perceptions.

#### Limitations

The study involved exclusively dental students in the pre-clinical phase of their studies. The results may not be generalisable to advanced or clinical training phases. Furthermore, participation in the study was voluntary, which suggests the presence of selection bias. Allocation to the IG and CG was not randomised, which constitutes a further limitation. A limitation is also the fact that the CG and IG had different sizes.

In this context, it should be acknowledged that assessing baseline comparability between groups is a retrospective verification rather than a feature of the study design. Therefore, it cannot fully compensate for the lack of randomisation, constituting an epistemological limitation that cannot be remedied retrospectively. Consequently, any assumptions regarding group equivalence at baseline should be treated with caution.

Only the students’ subjective perceptions were examined. The use of self-reported data leads to potential biases due to social desirability. Answers that are socially inappropriate tend not to be answered honestly and are therefore systematically over- or underestimated (61). Objective learning outcomes (e.g. examination results) were not measured. A comparison between the students’ subjective perceptions and changes in objective academic performance would be of interest.

Although both groups received the same total amount of instructional time (45 min), it was distributed differently (CG: 45-minute lecture; IG: 15-minute video preparation plus 30-minute interactive session). This represents a potential confounding factor, as the observed effects may be attributable not only to the ICM, but also to the greater proportion of structured, interactive time allocated to the IG. Therefore, it remains unclear whether the outcomes are driven by the pedagogical approach itself or by differences in time allocation and learning activities.

As the study was conducted at a single institution, external validity is limited. To ensure the generalisability of the results, transferability to other sites within the framework of a multicentre study design would be conceivable.

The intervention period was relatively short, which limited the possibility of identifying long-term effects. From a theoretical perspective, the study mainly addresses Level 1 (“Reaction”) of the Kirkpatrick model, with only indirect indications of Level 2 (“Learning”) [51].

In addition, it should be considered that German language proficiency at the German Language Examination for University Entrance (DSH) 2 level is required for admission, which may reduce potential linguistic barriers but does not fully eliminate individual differences in comprehension and active participation.

#### Generalisability

Given these limitations, the findings should be interpreted with caution and considered preliminary. The results relate to a single course in the early stages of dental education at a single institution, and therefore reflect the implementation of the ICM in a specific context.

Accordingly, their transferability to other disciplines, educational levels, institutional settings or long-term educational outcomes remains uncertain. Further research across diverse contexts is required before any broader conclusions about the role of the ICM in dental education can be drawn.

### Implications for research and practice

The results suggest that the ICM can promote student engagement, interaction and flexibility. However, careful implementation is required, particularly in the early stages of study, to avoid overload and ensure appropriate support for self-directed learning. In particular, the findings regarding the impact on interest in the course highlight the risk of cognitive overload.

Future studies should incorporate objective outcome measures (e.g. performance data), use longitudinal designs, examine adaptation processes to the ICM, and explore optimal combinations of ICM and FT.

Blended learning approaches that integrate ICM elements appear promising but should be implemented with institutional support, including technical infrastructure and pedagogical training for teaching staff.

## Conclusion

Within the specific context of preclinical dental education at a single German university, the ICM was associated with more favorable student ratings of preparation, flexibility, collaborative learning, teacher engagement, and active participation compared to traditional lectures over a 12-week period. However, no differences were observed in study interest or self-reported learning strategies, and the study may have been insufficiently sensitive to detect small effects. Overall, these findings suggest that ICM may complement rather than replace traditional teaching approaches. Interpretation is limited by the absence of random assignment, low internal consistency for some measures, and the lack of objective learning outcome data. Future research should employ randomized designs, longer intervention periods, and performance-based assessments.

## Supplementary Information

Supplementary Material 1.

Supplementary Material 2.

## Data Availability

The datasets used and analysed during the current study are available from the corresponding author on reasonable request.

## Abbreviations

BEvaKomp: Berlin evaluation tool for self-assessed student competencies
CG: Control group
D: Effect size
DSH: German Language Examination for University Entrance
EvaSys: Electric paper evaluationsystems
FLIPPY: Questionnaire for evaluating flipped classroom courses
FSI: Questionnaire on study interests
FT: Frontal teaching
IG: Intervention group
ICM: Inverted classroom model
LIST-K: Short version of the questionnaire on learning strategies at university
M: Mean value
N: Number
R: Rosenthal’s r
SD: Standard deviation
Q: Question
ZQ: Center for Quality Assurance & Development
