# Supplementary material for: A comparison of student evaluations of teaching and learning in the inverted classroom model versus traditional lectures in dental education
Source: BMC Med Educ. 2026 Jun 20;26:1015. doi: 10.1186/s12909-026-09733-3 (PMC13285014; doi:10.1186/s12909-026-09733-3)
Supplement: Supplementary file 1 — Supplementary Material 1. [file 12909_2026_9733_MOESM1_ESM.docx]

# Supplementary file

## Supplementary file 1: statistical comparison of socio-demographic data

| Feature | Characteristic | IG | CG | P value |
| --- | --- | --- | --- | --- |
| Age | years | 21.22 (SD ± 2,743) | 20.93 (SD ± 2,367) | 0.758 |
| Gender | male | 13 | 23 | 0.918 |
|  | female | 42 | 60 |  |
|  | divers | 0 | 0 |  |
| Mother tongue | German | 44 | 69 | 0.320 |
|  | other | 11 | 14 |  |
| Previous education | medical/dental/dental technician training | 16 | 15 | 0.152 |
|  | university degree | 2 | 5 |  |
|  | none | 34 | 58 |  |
|  | other | 3 | 5 |  |
| Professional activity alongside studies | medical/dental/dental technology sector | 20 | 22 | 0.905 |
|  | non-medical sector | 7 | 21 |  |
|  | none | 28 | 40 |  |

## Supplementary file 2: Item overall statistics for the learning experience aspect of FLIPPY

| Question | Cronbach's alpha, if the item is omitted |
| --- | --- |
| 1.5. I feel like I learned a lot of new things at this course. | 0.360 |
| 1.6. I feel adequately prepared for the examination thanks to the course. | 0.427 |
| 1.7. I gained important transfer knowledge and a deeper understanding through the course. | 0.436 |
| 1.8. I learn superficially and by heart in the course. | 0.586 |
| 1.9. I find it difficult to follow the course continuously. | 0.675 |
| 1.10. I feel that I can effectively acquire knowledge in this course. | 0.371 |
| Cronbachs alpha | 0.542 |

## Supplementary file 3: Results of the FLIPPY questionnaire on the aspect: video

| **Aspect: video** | | | |
| --- | --- | --- | --- |
| Question | Teaching concept | M  IG | SD  IG |
| 1.30. The videos help me to acquire new knowledge effectively. | ICM | 3.97 | 0.928 |
| 1.31. The videos are well designed and easy to understand in terms of content. | ICM | 3.83 | 0.950 |
| 1.32. Watching the videos makes me feel well prepared for upcoming events. | ICM | 3.73 | 1.202 |
| 1.33. I am interested in the content of the videos. | ICM | 4.30 | 0.702 |
| 1.34. Providing the learning content in the form of videos seems sensible to me. | ICM | 4.20 | 0.961 |
| 1.35. I watch the videos regularly. | ICM | 4.23 | 1.104 |
| Score_Video | ICM | 4.04 | 0.655 |

## Supplementary file 4: Results of the questionnaire on study interest (FSI)

| Question | Group | M  (t0) | SD (t0) | M (t1) | SD (t1) | p-value  Wilcoxon (two-  sided ) | p-value MWU  (two-  sided) |
| --- | --- | --- | --- | --- | --- | --- | --- |
| 2.1. I am sure that I have chosen the subject that suits my personal inclinations. | IG | 2.71 | 0.469 | 2.64 | 0.633 | 0.317 | 0.910 |
|  | KG | 2.63 | 0.667 | 2.60 | 0.545 | 0.782 |  |
| 2.2. After a long weekend or vacation, I look forward to studying again. | IG | 2.21 | 0.426 | 2.14 | 0.663 | 0.705 | 0.827 |
|  | KG | 2.18 | 0.594 | 2.05 | 0.639 | 0.166 |  |
| 2.3. If I had enough time, I would deal more intensively with certain questions related to my studies, even independently of exam requirements. | IG | 2.14 | 0.663 | 1.86 | 0.535 | 0.157 | 0.530 |
|  | KG | 2.18 | 0.675 | 2.07 | 0.694 | 0.502 |  |
| 2.4. I am sure that studying this subject will have a positive influence on my personality. | IG | 2.57 | 0.514 | 2.57 | 0.646 | 1.000 | 0.420 |
|  | KG | 2.53 | 0.599 | 2.35 | 0.700 | 0.191 |  |
| 2.5. For me, studying the content of my subject has actually very little to do with self-fulfillment. | IG | 2.43 | 0.646 | 2.29 | 0.611 | 0.564 | 0.902 |
|  | KG | 2.44 | 0.641 | 2.35 | 0.662 | 0.360 |  |
| 2.6. When I am in a library or bookstore, I like to browse through magazines or books that address topics related to my field of study, I also enjoy reading books that are not related to my studies, such as novels or biographies. | IG | 1.57 | 0.646 | 1.43 | 0.938 | 0.480 | 0.855 |
|  | KG | 1.52 | 0.816 | 1.40 | 0.955 | 0.403 |  |
| 2.7. I prefer to talk about my hobbies rather than my field of study. | IG | 1.50 | 0.650 | 1.79 | 0.579 | 0.257 | 0.222 |
|  | KG | 1.7 | 0.723 | 1.58 | 0.675 | 0.290 |  |
| 2.8. It was of great personal importance to me to be able to study this particular subject. | IG | 2.50 | 0.650 | 2.50 | 0.760 | 1.000 | 0.428 |
|  | KG | 2.75 | 0.543 | 2.63 | 0.667 | 0.096 |  |
| 2.9. I chose my current course of study primarily because of the interesting subject matter. | IG | 2.29 | 0.726 | 2.50 | 0.519 | 0.180 | 0.177 |
|  | KG | 2.43 | 0.747 | 2.38 | 0.774 | 0.617 |  |
| 2.10. Even before I started my studies, the subject I am now studying was very important to me, I have always been interested in the field of study. | IG | 1.79 | 0.802 | 1.79 | 0.699 | 1.000 | 0.572 |
|  | KG | 2.35 | 0.864 | 2.23 | 0.832 | 0.282 |  |
| 2.11. Compared to other things that are very important to me (e.g., hobbies, social relationships), I tend to attach little importance to my studies, and I am not very interested in my studies. | IG | 2.71 | 0.469 | 2.43 | 0.514 | 0.046* | 0.053 |
|  | KG | 2.50 | 0.506 | 2.57 | 0.594 | 0.439 |  |
| 2.12. Engaging with certain topics has a positive effect on my mood. | IG | 2.14 | 0.770 | 1.85 | 0.899 | 0.160 | 0.462 |
|  | KG | 1.85 | 0.700 | 1.70 | 0.823 | 0.235 |  |
| 2.13. To be honest, I sometimes feel rather indifferent about my field of study. | IG | 2.71 | 0.469 | 2.57 | 0.646 | 0.414 | 0.866 |
|  | KG | 2.75 | 0.439 | 2.63 | 0.667 | 0.059 |  |
| 2.14. Studying specific course content is more important to me than distractions, leisure time, and entertainment. | IG | 1.57 | 0.646 | 1.71 | 0.825 | 0.527 | 0.924 |
|  | KG | 1.45 | 0.783 | 1.53 | 0.751 | 0.567 |  |
| 2.15. Many areas of my field of study leave me feeling indifferent. | IG | 1.86 | 0.663 | 1.71 | 0.611 | 0.480 | 0.504 |
|  | KG | 2.00 | 0.847 | 1.77 | 0.620 | 0.123 |  |
| 2.16. Dealing with the content and problems of my field of study is not exactly one of my favorite activities. | IG | 2.29 | 0.611 | 1.86 | 0.770 | 0.014* | 0.110 |
|  | KG | 2.15 | 0.736 | 2.05 | 0.597 | 0.400 |  |
| 2.17. Even before starting my studies, I voluntarily engaged with content related to my field of study (e.g., reading books, attending lectures, having discussions). | IG | 1.79 | 0.699 | 1.43 | 0.646 | 0.132 | 0.856 |
|  | KG | 1.85 | 0.864 | 1.53 | 0.987 | 0.016* |  |
| 2.18. I rarely enjoy talking about the content of my studies. | IG | 2.64 | 0.497 | 2.50 | 0.519 | 0.317 | 0.991 |
|  | KG | 2.65 | 0.622 | 2.52 | 0.554 | 0.302 |  |

## Supplementary file 5: Results of the questionnaire on learning strategies during studies (LIST-K)

| Question | Group | M  (t0) | SD (t0) | M (t1) | SD (t1) | p-value  Wilcoxon (two-  sided ) | p-value MWU  (two-  sided) |
| --- | --- | --- | --- | --- | --- | --- | --- |
| 3.1. I go through my notes and make an outline with the most important points. | IG | 3.79 | 0.579 | 3.57 | 1.342 | 0.577 | 0.816 |
|  | KG | 3.55 | 1.037 | 3.35 | 1.292 | 0.386 |  |
| 3.2. I compile short summaries of the main ideas from my notes, scripts or literature. | IG | 4.36 | 0.745 | 4.21 | 1.122 | 0.414 | 0.444 |
|  | KG | 3.65 | 1.231 | 3.58 | 1.259 | 0.755 |  |
| 3.3. I compile important technical terms and definitions in separate lists. | IG | 3.71 | 1.069 | 3.86 | 1.099 | 0.564 | 0.171 |
|  | KG | 3.48 | 1.301 | 3.18 | 1.238 | 0.187 |  |
| 3.4. I try to relate new concepts or theories to concepts and theories I am already familiar with. | IG | 4.00 | 0.961 | 3.79 | 0.893 | 0.317 | 0.577 |
|  | KG | 3.68 | 0.859 | 3.63 | 1.005 | 0.845 |  |
| 3.5. I come up with specific examples learning content. | IG | 3.14 | 1.027 | 2.86 | 1.099 | 0.557 | 0.869 |
|  | KG | 3.30 | 1.114 | 3.33 | 1.047 | 0.964 |  |
| 3.6. I relate what I learn to my own experiences. | IG | 3.79 | 1.311 | 4.00 | 0.877 | 0.659 | 0.558 |
|  | KG | 3.78 | 1.025 | 3.93 | 0.917 | 0.441 |  |
| 3.7. I wonder if the text I am currently working on is really convincing. | IG | 2.93 | 1.141 | 2.57 | 0.852 | 0.281 | 0.095 |
|  | KG | 2.80 | 1.159 | 3.05 | 1.197 | 0.161 |  |
| 3.8. I approach most texts critically. | IG | 2.79 | 1.122 | 2.50 | 1.019 | 0.285 | 0.246 |
|  | KG | 2.90 | 1.172 | 3.00 | 1.155 | 0.460 |  |
| 3.9. I also critically examine what I learn. | IG | 2.79 | 1.424 | 2.71 | 1.267 | 0.773 | 0.722 |
|  | KG | 3.23 | 1.187 | 3.30 | 0.911 | 0.545 |  |
| 3.10. I memorize a self-created overview of the most important technical terms. | IG | 4.36 | 0.842 | 4.14 | 1.027 | 0.518 | 0.701 |
|  | KG | 3.74 | 1.117 | 3.63 | 1.192 | 0.624 |  |
| 3.11. I memorize rules, technical terms, or formulas. | IG | 4.50 | 0.650 | 4.71 | 0.469 | 0.317 | 0.186 |
|  | KG | 4.30 | 0.723 | 4.15 | 0.736 | 0.269 |  |
| 3.12. I learn the material from scripts or other notes memorizing it as much as possible. | IG | 4.43 | 0.852 | 3.71 | 1.139 | 0.031* | 0.080 |
|  | KG | 3.78 | 0.768 | 3.75 | 1.056 | 0.948 |  |
| 3.13 I formulate learning objectives, which I then use to guide my learning. | IG | 2.79 | 1.311 | 3.00 | 1.177 | 0.655 | 0.507 |
|  | KG | 2.83 | 1.394 | 2.83 | 1.375 | 0.904 |  |
| 3.14. Before studying, I think about how I want to study. | IG | 4.00 | 1.038 | 4.07 | 0.616 | 0.791 | 0.927 |
|  | KG | 3.60 | 1.150 | 3.55 | 1.239 | 0.803 |  |
| 3.15. I do not plan my approach to learning. | IG | 4.31 | 0.751 | 4.07 | 0.917 | 0.366 | 0.305 |
|  | KG | 3.98 | 1.074 | 3.40 | 1.236 | < 0.001* |  |
| 3.16. To identify gaps in my knowledge, I recap the most important content without referring to my documents. | IG | 3.21 | 1.424 | 3.43 | 1.284 | 0.405 | 0.959 |
|  | KG | 3.43 | 1.174 | 3.70 | 1.114 | 0.262 |  |
| 3.17. I ask myself questions about the material to check whether I have understood everything. | IG | 3.57 | 1.222 | 3.93 | 0.829 | 0.339 | 0.465 |
|  | KG | 3.68 | 0.859 | 3.90 | 0.841 | 0.101 |  |
| 3.18. If the learning material contains questions or tests, I use them to test myself. | IG | 4.86 | 0.363 | 4.57 | 0.646 | 0.102 | 0.840 |
|  | KG | 4.50 | 0.641 | 4.35 | 0.662 | 0.291 |  |
| 3.19. I change my learning technique when I encounter difficulties. | IG | 3.21 | 1.051 | 3.71 | 1.069 | 0.070 | 0.310 |
|  | KG | 3.38 | 1.102 | 3.48 | 0.933 | 0.550 |  |
| 3.20. I change my learning plans when I realize that they cannot be implemented. | IG | 3.64 | 0.842 | 4.00 | 0.679 | 0.218 | 0.185 |
|  | KG | 3.80 | 0.939 | 3.65 | 1.099 | 0.537 |  |
| 3.21. When I realize that my approach to learning is not successful, I change it. | IG | 4.00 | 0.679 | 3.79 | 0.893 | 0.429 | 0.371 |
|  | KG | 3.68 | 0.971 | 3.75 | 0.899 | 0.714 |  |
| 3.22. I find it difficult to stay focused. | IG | 3.43 | 0.938 | 3.07 | 1.328 | 0.129 | 0.620 |
|  | KG | 3.33 | 1.047 | 2.85 | 1.292 | 0.026* |  |
| 3.23. I have trouble concentrating when studying. | IG | 3.71 | 0.994 | 3.36 | 1.216 | 0.132 | 0.728 |
|  | KG | 3.50 | 0.987 | 3.23 | 1.165 | 0.047* |  |
| 3.24. When I study, I am easily distracted. | IG | 3.36 | 1.277 | 3.07 | 1.385 | 0.431 | 0.352 |
|  | KG | 3.08 | 1.023 | 2.88 | 1.244 | 0.162 |  |
| 3.25. When I set myself a specific amount of work to study, I make an effort to complete it. | IG | 4.36 | 0.497 | 4.36 | 0.633 | 1.000 | 0.485 |
|  | KG | 4.38 | 0.667 | 4.22 | 0.698 | 0.109 |  |
| 3.26. I do not give up, even if the material is very difficult or complex. | IG | 4.00 | 0.961 | 3.93 | 0.997 | 0.705 | 0.722 |
|  | KG | 4.15 | 0.700 | 4.10 | 0.955 | 0.826 |  |
| 3.27. I also study late in the evening and on weekends if necessary. | IG | 4.43 | 0.756 | 4.43 | 0.852 | 1.000 | 0.774 |
|  | KG | 4.25 | 0.840 | 4.38 | 0.868 | 0.337 |  |
| 3.28. When studying, I stick to a specific schedule. | IG | 3.3 | 1.008 | 3.14 | 1.099 | 0.317 | 0.356 |
|  | KG | 3.03 | 1.271 | 3.08 | 0.997 | 0.707 |  |
| 3.29. I set the hours I spend studying each day by making a schedule. | IG | 2.07 | 0.997 | 2.57 | 1.222 | 0.161 | 0.055 |
|  | KG | 2.25 | 1.276 | 2.05 | 1.280 | 0.176 |  |
| 3.30. I set a specific time period before each learning phase. | IG | 2.71 | 1.267 | 2.57 | 0.938 | 0.628 | 0.715 |
|  | KG | 2.53 | 1.358 | 2.58 | 1.357 | 0.769 |  |
| 3.31. I work on texts or assignments together with my fellow students. | IG | 3.07 | 0.997 | 3.0 | 1.072 | 1.000 | 0.772 |
|  | KG | 2.65 | 1.075 | 2.60 | 1.194 | 0.684 |  |
| 3.32. I take time to discuss the material with fellow students. | IG | 3.57 | 0.938 | 3.50 | 1.019 | 0.783 | 0.687 |
|  | KG | 3.25 | 1.056 | 3.20 | 1.265 | 0.748 |  |
| 3.33. If something is not clear to me, I ask a fellow student for advice. | IG | 4.43 | 0.646 | 4.29 | 0.611 | 0.480 | 0.369 |
|  | KG | 4.15 | 0.802 | 4.18 | 0.931 | 0.745 |  |
| 3.34. I look for further reading when certain topics are not totally clear to me. | IG | 4.00 | 0.784 | 4.07 | 0.730 | 0.763 | 0.141 |
|  | KG | 4.00 | 0.877 | 3.55 | 1.319 | 0.044* |  |
| 3.35. I gather missing information from various sources (e.g., lecture notes, books and journals). | IG | 4.14 | 0.864 | 4.21 | 0.802 | 0.527 | 0.079 |
|  | KG | 4.13 | 1.017 | 3.65 | 1.122 | 0.029* |  |
| 3.36. I consult additional literature if my notes are incomplete. | IG | 4.00 | 1.038 | 4.00 | 0.784 | 0.914 | 0.383 |
|  | KG | 4.23 | 0.733 | 3.85 | 0.949 | 0.008* |  |
| 3.37. I organize my environment so that I am distracted as little as possible from learning. | IG | 4.14 | 1.027 | 4.21 | 0.975 | 0.564 | 0.825 |
|  | KG | 3.70 | 0.911 | 3.78 | 0.974 | 0.600 |  |
| 3.38. I always sit in the same place when I study. | IG | 3.57 | 1.222 | 3.79 | 1.122 | 0.366 | 0.491 |
|  | KG | 3.48 | 1.358 | 3.48 | 1.320 | 1.000 |  |
| 3.39. My workplace is designed so that I can find everything quickly. | IG | 4.14 | 0.864 | 4.29 | 0.914 | 0.623 | 0.762 |
|  | KG | 4.25 | 0.840 | 4.28 | 0.877 | 0.819 |  |

## Supplementary file 6: Personal attitude towards teaching

| Question | Group | M (t0) | SD (t0) | M (t1) | SD (t1) | p-value  Wilcoxon  (two-  sided ) | p-value MWU  (two-  sided) |
| --- | --- | --- | --- | --- | --- | --- | --- |
| 4.1. I believe that teaching should be adapted to developments and advances over time. | IG | 3.50 | 0.760 | 3.50 | 0.519 | 1.000 | 0.723 |
|  | KG | 3.58 | 0.636 | 3.50 | 0.555 | 0.513 |  |
| 4.2. I am open to new teaching methods and approaches to teaching the subject matter. | IG | 3.14 | 0.864 | 3.43 | 0.646 | 0.157 | 0.377 |
|  | KG | 3.20 | 0.823 | 3.28 | 0.716 | 0.491 |  |
| 4.3. I think face-to-face teaching is important in our degree program and I prefer it. | IG | 3.86 | 0.363 | 3.36 | 0.497 | 0.020* | 0.005* |
|  | KG | 3.38 | 0.774 | 3.43 | 0.813 | 0.593 |  |
| 4.4. I prefer digital teaching. | IG | 2.00 | 0.679 | 2.14 | 0.363 | 0.414 | 0.893 |
|  | KG | 2.05 | 0.749 | 2.20 | 0.992 | 0.196 |  |
| 4.5. In my opinion, digital teaching should be combined with face-to-face teaching. | IG | 3.21 | 0.893 | 3.43 | 0.646 | 0.429 | 0.310 |
|  | KG | 3.23 | 0.891 | 3.15 | 1.027 | 0.682 |  |

## Supplementary file 7: Item overall statistics for personal attitude towards teaching

| Question | Cronbach's alpha, if the item is omitted |
| --- | --- |
| 4.1. I believe that teaching should be adapted to developments and advances over time. | -0.183 |
| 4.2. I am open to new teaching methods and approaches to teaching the subject matter. | -0.182 |
| 4.3. I think face-to-face teaching is important in our degree program and I prefer it. | 0.553 |
| 4.4. I prefer digital teaching. | 0.218 |
| 4.5. In my opinion, digital teaching should be combined with face-to-face teaching. | -0.128 |
| Cronbachs alpha | 0.174 |
